# In Memoriam—Rev. C. P. Jennings, M. D.

**Published:** 1895-01

**Authors:** 


### IN MEMORIAM—REV. C. P. JENNINGS, S. T. D., M. D.

Resolutions passed by the Central New York Homœopathic Medical Society, at Rochester, N. Y., December 20th, 1894:

As the years roll by we are painfully reminded of the brevity and uncertainty of life. From time to time we have been called upon to mourn the loss of some one of our members, until they who have gone before us number as many as those who yet live and act with us. Glancing backward, we recall the names of Drs. Clary, Hawley, Hoyt, Miller, J. G. Bigelow, Frank Bigelow, Smith, Potter, Pool, Spooner, Benson, Sumner, Gardner, Wells, Mera, Schenck, Brown, T. L. Brown, Munger, Robinson, Squier, Richards, Schmitt, and now we learn of the death of one of our old Secretaries, Rev. C. P. Jennings, S. T. D., M. D., who died at his daughter's residence in Shelbyville,

Ind., November 20th, 1894. He was Secretary of this Society from December 18th, 1879, to December 20th, 1883.

Dr. Jennings was an Episcopal clergyman who officiated at several points in this State, a man of decided ability, great decision of character, fearless in his espousal of the right as he recognized it, a warm adherent of Hahnemannian Homœopathy, and a zealous advocate of it. Of late years his painful infirmity, multiple sclerosis, cut him off from intercourse with us, and prevented his active participation in the work, but we feel assured that he was with us in spirit. We deem it not only well, but a duty to record and publish these sentiments in a suitable way, and to that end we offer the following:

WHEREAS, Death has again taken one of our number in the person of Rev. C. P. Jennings, S. T. D., M. D., a time-honored, respected, and zealous member, formerly Secretary of this Society, therefore,

*Resolved*, That we fully understand and acknowledge the meaning of the loss of such members of this Society as Dr. C. P. Jennings, who was a scholar, a man of integrity, an uncompromising homœopathician.

*Resolved*, That in the capacity of Secretary of the Central New York Homœopathic Medical Society during the last year of the seventh and the first years of the eighth decade of this century, he rendered us very valuable service, for which we are under obligation to his memory, which we cherish.

*Resolved*, That we truly sympathize with his widow, if she survives him, and with his relatives and friends, wherever they may be, in their bereavement, and we rejoice with them in the fact that he maintained an honorable, just, and consecrated life, a life devoted to the welfare of humanity.

*Resolved*, That a copy of these resolutions be furnished the family of Dr. Jennings, and to THE HOMŒOPATHIC PHYSICIAN and the *Medical Advance* for publication.

T. DWIGHT STOW,
A. B. CARR,
*Committee.*

